# P05.60. Dragon boat racing: an evaluation of its influence on the health-related quality of life of breast cancer survivors

**DOI:** 10.1186/1472-6882-12-S1-P420

**Published:** 2012-06-12

**Authors:** H Ray, M Verhoef

**Affiliations:** 1 Mount Royal University, Calgary, Alberta, Canada; 2 University of Calgary, Calgary, Canada

## Purpose

The purpose of this study was to: 1) determine whether and how breast cancer survivors’ participation in a season of dragon boat racing influenced health-related quality of life (HRQOL). And if so, to what degree changes are reflected within the physical, emotional, social/family and spiritual domains of HRQOL, 2) explore the breast cancer survivor experience of dragon boating and how and why this experience is perceived to influence HRQOL.

## Methods

A mixed methods sequential explanatory design was used to examine the relationship between dragon boat racing and HRQOL of breast cancer survivors. One hundred women completed on-line surveys at baseline and post-season periods. Four measures from the Functional Assessment of Chronic Illness Therapy (FACIT) measurement system were employed to measure HRQOL. From the sample, 15 women were selected for an interview at the end of the season to obtain a deeper understanding of the lived experience of dragon boat racing.

## Results

Statistically significant improvements from early to late season were reported for HRQOL, physical and emotional well-being, breast cancer-specific concerns and cancer-related fatigue. A trend towards significance was reported for functional well-being, with improved social/family and spiritual well-being scores indicated post-season. Qualitative data elaborated on the quantitative findings, greatly enhancing the understanding of how and why dragon boat racing influenced HRQOL of participating breast cancer survivors.

## Conclusion

Participation in a season of dragon boat racing is associated with improvements in HRQOL, physical and emotional well-being, breast cancer-specific concerns and cancer-related fatigue. Enhanced social/family, functional and spiritual well-being of participating breast cancer survivors was also indicated. These findings contribute to a growing literature supporting the benefits of dragon boat racing, bringing breast cancer survivors another step closer to having a physical activity option that may help them address their HRQOL challenges, while encouraging them to thrive during survivorship.

